# Late Presentation of Hirayama Disease With “Snake Eye Sign”: A Case Report

**DOI:** 10.7759/cureus.21557

**Published:** 2022-01-24

**Authors:** Sarvesh C Mishra, Vivek Singh, Anil K Singh, Srishti Sharma, Isha Tyagi

**Affiliations:** 1 Radiodiagnosis, Sanjay Gandhi Postgraduate Institute of Medical Sciences, Lucknow, IND; 2 Neuro-otology, Sanjay Gandhi Postgraduate Institute of Medical Sciences, Lucknow, IND

**Keywords:** hirayama disease, young males, monomelic amyotrophy, snake eye appearance, cervical myelopathy

## Abstract

Hirayama disease, also called non-progressive juvenile muscular atrophy of distal upper limbs, is a type of cervical myelopathy associated with flexion movements of the neck. It is a type of benign motor neuron disease seen typically in young males in the age group of 15 to 25. The disease has an insidious onset with a stationary stage following a progressive phase. It is also called monomelic amyotrophy with patients usually presenting with insidious onset unilateral upper limb weakness and muscle wasting. A bilateral and asymmetrical presentation can be seen very rarely.

A middle-aged male patient presented with bilateral asymmetrical upper limb weakness, muscle wasting involving forearm and hand muscles. Neurological examination showed bilateral upper limb weakness and muscle wasting involving forearm and hand muscles, with a classical pattern of muscle wasting in bilateral forearm muscles called oblique amyotrophy. A clinical diagnosis of Hirayama disease was made and the patient was sent to the radiology department for Magnetic Resonance Imaging of the cervical spine in flexion and neutral positions. The imaging findings were consistent with the clinical diagnosis of Hirayama disease with the presence of an abnormal “snake eye appearance”. The electrophysiological assessment done including the electromyography and nerve conduction studies were also consistent with the clinical diagnosis.

“Snake eye appearance” on MRI in patients with Hirayama disease is associated with unfavorable outcomes and represents cervical myelopathy involving the anterior horn cells.

## Introduction

Hirayama disease is an uncommon neurological entity and is a type of benign motor neuron disease. The patients are usually young males presenting with insidious onset of unilateral upper limb weakness with associated muscle wasting in the forearm and hand [1]. The disease affects the C7, C8, and T1 myotomes. There is usually no sensory involvement or pyramidal tract involvement. Magnetic Resonance Imaging (MRI) cervical spine in flexion and extension are the mainstay for radiological diagnosis showing prominent epidural space due to detachment of the posterior dura mater from the underlying lamina. The prominent posterior epidural space can show post-contrast enhancement. Occasionally, prominent epidural flow voids are also seen due to the epidural vessels traversing the space. In the cervical spine, the lower cervical spinal cord is usually affected and shows focal atrophy and cord hyperintensity. 

Electrophysiological studies form an important part of the diagnostic battery in cases of Hirayama disease. Jin X. et al [2] in their study demonstrated that the ulnar/median compound muscle action potential (CMAP) ratio was lower in Hirayama disease (0.55 ± 0.41, P<0.0001), higher in amyotrophic lateral sclerosis (ALS) (2.28 ± 1.15, P<0.0001), and no different in cervical spondylotic amyotrophy (CSA) (1.21 ± 0.53, P>0.05) compared with the normal range from previous studies (0.89-1.60) and with the healthy controls (1.15 ± 0.23). Conduction velocities of the sensory and motor nerves, the amplitude of the sensory nerve action potential, and the CMAP amplitude of the unaffected limb were all normal.

Snake-eyes appearance (SEA) is an MR imaging finding characterized by symmetrical bilateral small hyperintense lesions on an axial T2-weighted MRI having a similar appearance to the face of a snake. It is also called as "owl-eye sign" or "fried-eggs sign".

It is suggested by some authors to be a reversible change, akin to edema or gliosis [3-5]. However, pathologically it represents cystic necrosis at the junction of the central gray matter near the ventrolateral posterior column [6]. 

Hirayama disease is known to be a self-limited disease. However, Xu H. et al in their study concluded that Hirayama disease has the possibility of progressing to cervical spondylosis [7]. They also observed that patients with SEA on MRI were more likely to show pyramidal signs and progression to cervical spondylosis which is not a self-limiting disease unlike Hirayama disease and is associated with poor outcomes.

## Case presentation

A 45-year-old male patient presented with complaints of weakness in bilateral upper limbs. These changes were asymmetric with the involvement of the left upper limb more marked than the right upper limb. The symptoms were insidious in onset and gradual in progression initially starting in the left upper limb and later involving the right upper limb. The symptoms were aggravated on neck flexion. On examination, there was marked wasting of both forearm and hand muscles bilaterally. There was atrophy of muscles of the bilateral forearm with sparing of the bulk of brachioradialis muscle with a classical appearance of "oblique amyotrophy". The left upper extremity strength was 5/5 except, hand grip was 2/5, finger adduction and abduction were 2/5, and dorsiflexion of the wrist and fingers was 2/5. . The right upper extremity strength was 5/5 except, hand grip was 3/5, finger adduction and abduction were 3/5, and dorsiflexion of the wrist and fingers was 3/5.

Fasciculations, mild atrophy of the forearm, and atrophy of the intrinsic hand muscles as well as both thenar and hypothenar muscles (with no evidence of split hand sign) were seen bilaterally. Strength was 5/5 throughout lower extremities. Reflexes were 3+/4 in biceps, triceps, and brachioradialis bilaterally; and 3+/4 in bilateral knees and ankles. Hoffman and Babinski signs were present bilaterally. Sensory modalities such as a pinprick, vibration, and tactile sensation were equal and intact in both arms and legs. Fine motor functions and grasp were limited by the weakness of extensors and flexors of the forearm and digits. Higher mental functions and cranial nerves examination were unremarkable. Pulses were present and limbs were warm with a good capillary refill. Vitamin B12 levels (383pg/ml) were within normal limits. His blood sugar levels were normal with fasting blood sugar (FBS) of 88mg/dl, the postprandial blood sugar level of 133mg/dl, and glycated hemoglobin (HbA1c) of 5.2%. 

**Figure 1:**
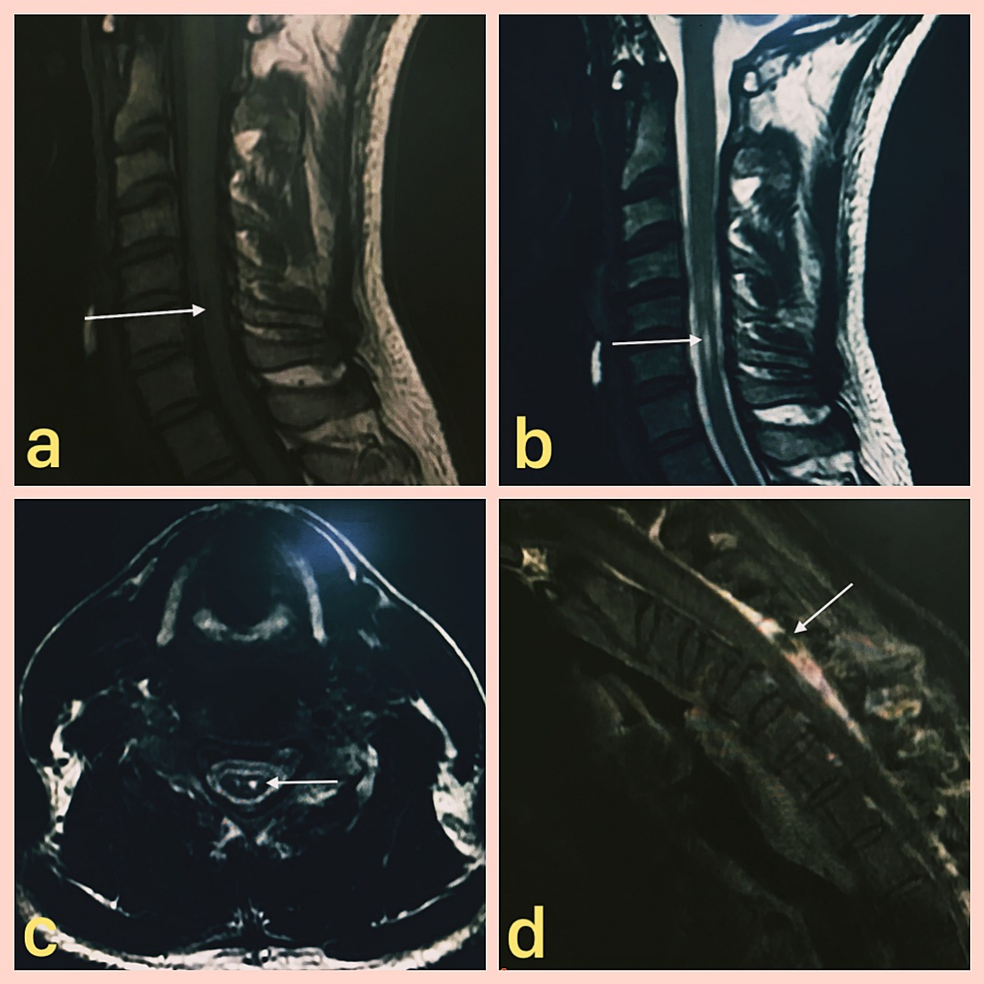
Contrast MRI of the cervicodorsal spine in neutral and flexion 1a. Sagittal T1-weighted image of the cervical spine showing linear hypointensity in the anterior portion of the lower cervical spinal cord (denoted by white arrow) with associated cord thinning. 1b. Sagittal T2-weighted image in neutral position showing linear hyperintense signal (denoted by white arrow) in the anterior portion of the cervical spinal cord with associated cord thinning. 1c. Axial T2-weighted image at the level of the lower cervical spine showing hyperintense signal in bilateral anterior horn cells giving a “Snake Eye Appearance”. 1d. Sagittal T1 post-contrast image in flexion shows prominent enhancing posterior epidural space with a flow void (denoted by white arrow).

A clinical diagnosis of Hirayama disease was made and a contrast MRI of the cervicodorsal spine in neutral and flexion was done which showed an abnormal linear T1 hypo-intense (Figure 1a) and T2 hyperintense signal (Figure 1b) in the anterior spinal cord with “Snake Eye Appearance” (SEA) on axial T2-weighted image (Figure 1c). There was an anterior displacement of the posterior dura mater anteriorly in flexion with prominent posterior epidural space showing post-contrast enhancement (Figure 1d). There was also a prominent flow void within the epidural space which is known to be seen in the Hirayama disease (Figure 1d). Thus the imaging findings were also consistent with the clinical diagnosis. The electrophysiological examination was done which showed decreased median and ulnar compound muscle action potential, standard sensory nerve conduction study, and features of denervation in the form of fasciculations and fibrillations. A neurogenic pattern was seen in C7, C8, and T1 myotomes on electromyography. The findings on the electrophysiological examination were also consistent with the clinical diagnosis. The patient was managed conservatively. 

He was asked to wear a cervical collar to stabilize the neck to reduce movements aggravating the symptoms. He got relief from the symptoms due to the flexion movements of the neck. However, the patient developed pyramidal signs over a period of the next 3 months and developed symptoms of cervical spondylosis.

## Discussion

Hirayama disease is a rare variety of cervical myelopathy typically affecting young males with symptoms aggravating on flexion of the spine. The disease manifests clinically as the insidious onset of unilateral upper limb weakness which is progressive initially and then stabilizes. Bilateral upper limb involvement is also reported when it is usually asymmetrical. There are no sensory or pyramidal signs. MRI is the main imaging modality for diagnosis with imaging done in flexion and extension. The usual imaging findings seen are prominent enhancing posterior epidural space in the cervical region, focal lower cervical spinal cord atrophy with hyperintense signal in cord parenchyma, flattening of the cervical cord, loss of normal cervical lordotic curvature, flow voids in the posterior epidural space, etc.

“Snake eye appearance” (SEA) is another uncommon finding seen in Hirayama disease on axial MRI images. Jinkins et al reported it for the first time in 1986 [8]. It is also called the “owl-eye sign “or “fried egg appearance” and refers to the bilateral hyperintense, circular, or ovoid foci in anterior horn cells of the spinal cord on axial T2-weighted MRI. It represents the cystic ischemia in the anterior horn cells. The relevance of the sign in prognostication of the disease is debatable with differing results in various articles [3, 9]. SEA appearance has been described with several clinical conditions like anterior spinal artery ischemia [10], chronic compressive myelopathy [11], Hirayama disease [7, 12], amyotrophic lateral sclerosis [13], etc. 

It has been conjectured that it is a reversible condition [3]. It is contradictory to the fact that on histopathology there is cystic necrosis at the junction of central gray matter near the ventrolateral posterior column [6]. The majority of the available literature reports the “snake eye appearance” to have an unfavorable prognosis in case of degenerative cervical myelopathy. However, Fontanella et al in a case series and review of the literature published in 2020 have concluded that SEA represents a negative surgical prognosis sign in a minority (22-28%) of patients [14]. However, baseline neurological status remains crucial to determine patient outcomes.

## Conclusions

SEA represents an irreversible ischemic change in bilateral anterior horn cells occurring at the point of the impression of the cord against the dura and signifies a poor prognosis. It appears late in the clinical course of Hirayama disease and is closely related to pyramidal signs and spinal cord atrophy. Contrary to the conventional belief that the Hirayama disease is self-limiting and shows no sensory or pyramidal signs, it is more likely to show progression to spondylosis if it shows SEA on MRI.
